# Opening One's Eyes to Mosaicism in Progressive External Ophthalmoplegia

**DOI:** 10.1212/NXG.0000000000000202

**Published:** 2017-12-15

**Authors:** Ewen W. Sommerville, Rachel L. Jones, Steven A. Hardy, Emma L. Blakely, Angela Pyle, Andrew M. Schaefer, Patrick F. Chinnery, Douglass M. Turnbull, Gráinne S. Gorman, Robert W. Taylor

**Affiliations:** From the Wellcome Centre for Mitochondrial Research (E.W.S., R.L.J., S.A.H., E.L.B., A.M.S., D.M.T., G.S.G., R.W.T.), Institute of Neuroscience, The Medical School, Newcastle University, United Kingdom; Department of Molecular and Human Genetics (E.W.S.), Baylor College of Medicine, Houston, TX; NHS Highly Specialised Mitochondrial Diagnostic Laboratory (R.L.J., S.A.H., E.L.B., R.W.T.), Newcastle upon Tyne Hospitals NHS Foundation Trust, United Kingdom; Wellcome Centre for Mitochondrial Research (A.P.), Institute of Genetic Medicine, Newcastle University, United Kingdom; and Department of Clinical Neurosciences (P.F.C.), School of Clinical Medicine, and MRC Mitochondrial Biology Unit (P.F.C.), University of Cambridge, United Kingdom.

Autosomal dominant progressive external ophthalmoplegia (adPEO) is a mendelian disorder of mitochondrial DNA (mtDNA) maintenance characterized by restricted eye movements, ptosis, and skeletal muscle–restricted multiple mtDNA deletions.^1^ Dominantly inherited pathogenic variants of *TWNK* (GenBank: NM_021830), encoding twinkle helicase, an essential protein required to unwind mtDNA during replication, are among the most common cause of adult-onset PEO,^2,3^ with patients manifesting relatively indolent PEO phenotypes, often with proximal muscle weakness, ataxia, visual loss or impairment, and neurologic symptoms.^1^ However, after excluding pathogenic variants in known genes, the genetic etiology remains undetermined in approximately 50% of patients. We describe the diagnostic odyssey of a patient who presented in the fourth decade of life with PEO and harbored a mosaic known pathogenic *TWNK* variant, initially undetected by conventional diagnostic Sanger sequencing but identified using next-generating sequencing.

A 41-year-old woman initially presented with a 9-year history of progressive ptosis associated with intermittent diplopia. Clinical examination revealed bilateral, asymmetric ptosis (right > left), external ophthalmoplegia, mild facial weakness, and mild proximal myopathy (Medical Research Council 4/5). Family history was unremarkable. Initially, she was investigated for myasthenia gravis that proved negative (tensilon test, repetitive nerve stimulation, and acetylcholine receptor antibodies). Electromyography showed only slight myopathic changes; the muscle creatine kinase level was elevated at 994 U/L (range 0–160); and lactate levels were normal (1.1 mmol/L; range 0.7–2.1). Muscle biopsy showed 20% cytochrome *c* oxidase–deficient fibers, many of which showed increased succinate dehydrogenase reactivity, typical of “ragged-blue” fibers (figure, A). Southern blotting of muscle DNA revealed multiple mtDNA deletions (figure, B), which were also previously confirmed by a quantitative real-time PCR assay^4^; our patient corresponds to subject M1 in the original publication.^4^

**Figure:**
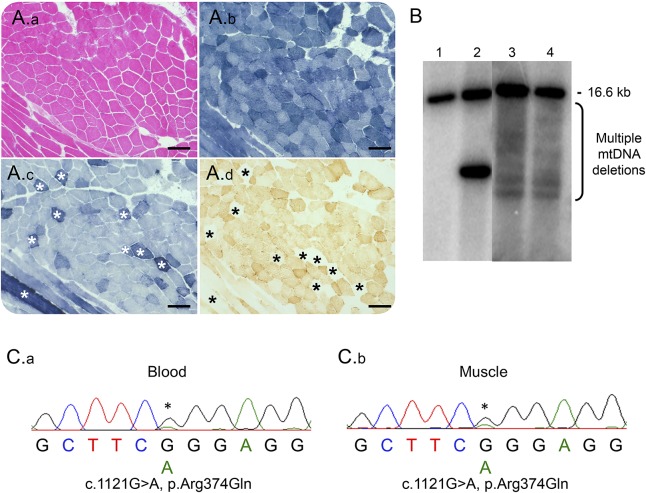
Histopathologic, molecular and genetic characterization of the mosaic c.1121G>A p.(Arg374Gln) *TWNK* variant (A) Diagnostic muscle biopsy was subjected to (A.a) H&E staining in addition to histochemical reactions for (A.b) NADH-tetrazolium reductase, (A.c) SDH, and (A.d) COX. Asterisk denotes COX-deficient, SDH-reactive fibers. Images were taken at ×10 magnification. Scale bar (solid black line) denotes 100 μM. (B) Southern blotting of skeletal muscle DNA using a PCR-generated D-loop (α-32P dCTP-labeled) probe, demonstrating multiple mtDNA deletions in the patient (lane 3). Also shown are wild-type DNA (lane 1), a patient with a single, large-scale mtDNA deletion (lane 2) and multiple mtDNA deletions in a patient harboring a pathogenic, heterozygous c.2864A>G p.(Tyr955Cys) *POLG* variant (lane 4). (C) Sanger sequencing electropherograms also suggested mosaicism in both (C.a) blood and (C.b) muscle DNA. COX = cytochrome *c* oxidase; SDH = succinate dehydrogenase.

Candidate screening of nuclear genes associated with PEO and multiple mtDNA deletions (*POLG*, *RRM2B*, *SLC25A4*, *TWNK*, *POLG2*, *TK2*, and *RNASEH1*) were apparently negative, prompting whole-exome sequencing with blood DNA. The mean depth per exome consensus coding sequence (CCDS) was 71-fold, while the mean percentage of CCDS bases at 20-fold coverage was 87.01%. Analysis of rare exonic variants in nuclear genes encoding mitochondrial-localized proteins or DNA transcription, replication, or maintenance machinery unexpectedly revealed a known pathogenic heterozygous c.1121G>A p.(Arg374Gln) *TWNK* variant.^2,3^ Analysis of additional genes associated with mtDNA maintenance disorders failed to detect pathogenic variants. Examination of read coverage at the c.1121 base (UCSC hg19, g.102749088) showed 30 reads that passed quality score filtering, with an unfiltered allele depth of 20 reads indicating wild-type and 10 reads indicating the variant (c.1121G>A, p.Arg374Gln) (figure e-1, http://links.lww.com/NXG/A8). Re-examination of the diagnostic electropherograms for *TWNK* from blood DNA revealed a small peak for the c.1121G>A p.(Arg374Gln) variant, which was confirmed by repeated Sanger sequencing of both blood and muscle DNA, suggestive of low-level mosaicism (figure, C). Targeted next-generation sequencing was subsequently performed to achieve a higher depth of coverage at the variant nucleotide and allow a more accurate estimation of the levels of mosaicism in these tissues. A read depth of >1,700 was observed in both tissues, and the levels of mosaicism were determined to be 13% in blood and 18% in muscle (figure e-2, http://links.lww.com/NXG/A9).

Somatic mosaicism is the phenomenon of a single variant occurring in 2 or more populations of soma cells in 1 individual and has been increasingly recognized as an important pathogenic mechanism in genetic disease.^5^ Probable germline mosaicism of dominant pathogenic *TWNK* and *SLC25A4* variants causing adult-onset PEO with multiple mtDNA deletions have been previously described.^6,7^ By contrast, we present evidence of somatic mosaicism for a pathogenic *TWNK* variant associated with this disorder. Due to the low level of mosaicism (18%) in skeletal muscle, we speculate that the variant may have arose during early embryogenesis,^5^ manifesting in a relatively indolent clinical phenotype. Despite low-level mosaicism, the variant could be highly detrimental to hexamer formation and helicase activity. Furthermore, diagnostic Sanger sequencing of *TWNK* initially failed to identify the mosaic variant, and hence, genetic analysis was reported as negative. While autosomal dominant disorders have a 50% recurrence risk in offspring, mosaicism also presents a considerable recurrence risk. For our patient, it was not possible to determine the extent of mosaicism in the germline, which provides further challenges for genetic counseling. Nonetheless, we provided a genetic diagnosis in a longstanding case, demonstrating the use of next-generation sequencing for detecting and quantifying low-level mosaicism.

In conclusion, we suggest that physicians should revisit suspected adPEO patients with apparently negative genetic testing of mtDNA maintenance disorder genes and to consider mosaicism. With expanded reproductive options and ongoing development of therapeutic strategies, our case highlights the importance of attaining a genetic diagnosis and the power of next-generation sequencing.
